# HIV-1 Genetic Variability and Clinical Implications

**DOI:** 10.1155/2013/481314

**Published:** 2013-06-17

**Authors:** Maria Mercedes Santoro, Carlo Federico Perno

**Affiliations:** ^1^Department of Experimental Medicine and Surgery, University of Rome Tor Vergata, Via Montpellier 1, 00133 Rome, Italy; ^2^INMI L Spallanzani Hospital, Antiretroviral Therapy Monitoring Unit, Via Portuense 292, 00149 Rome, Italy

## Abstract

Despite advances in antiretroviral therapy that have revolutionized HIV disease management, effective control of the HIV infection pandemic remains elusive. Beyond the classic non-B endemic areas, HIV-1 non-B subtype infections are sharply increasing in previous subtype B homogeneous areas such as Europe and North America. As already known, several studies have shown that, among non-B subtypes, subtypes C and D were found to be more aggressive in terms of disease progression. Luckily, the response to antiretrovirals against HIV-1 seems to be similar among different subtypes, but these results are mainly based on small or poorly designed studies. On the other hand, differences in rates of acquisition of resistance among non-B subtypes are already being observed. This different propensity, beyond the type of treatment regimens used, as well as access to viral load testing in non-B endemic areas seems to be due to HIV-1 clade specific peculiarities. Indeed, some non-B subtypes are proved to be more prone to develop resistance compared to B subtype. This phenomenon can be related to the presence of subtype-specific polymorphisms, different codon usage, and/or subtype-specific RNA templates. This review aims to provide a complete picture of HIV-1 genetic diversity and its implications for HIV-1 disease spread, effectiveness of therapies, and drug resistance development.

## 1. Introduction

Thirty years have passed after discovering human immunodeficiency virus (HIV), the etiological agent of the acquired immunodeficiency syndrome (AIDS) [1–4].

Two types of HIV are known: the most common HIV-1, which is responsible to the worldwide AIDS epidemic, and the immunologically distinct HIV-2 [5], which is much less common and less virulent [6, 7] but produces clinical findings similar to HIV-1 [8]. The HIV-1 type itself includes four groups M (main), O (outlier), N (non-M, non-O), and P [9–12], which have different geographic distributions but all produce similar clinical symptoms. The M group further splits into 9 subtypes (A through J) [13–15], as well as at least 58 circulating recombinant forms (CRFs, http://www.hiv.lanl.gov/content/sequence/HIV/CRFs/CRFs.html, last accessed 06 May 2013) and multiple unique recombinant forms (URFs).

The vast majority of reports on drug resistance deal with HIV-1 subtype B infections in developed countries, and this is largely due to historical delays in access to antiretroviral therapy on a worldwide basis.

Advances in antiretroviral therapy have revolutionized HIV management and the control of the spread of regional epidemics [16–18]. Currently, a combination of several antiretroviral agents, termed Highly Active Anti-Retroviral Therapy (HAART), has been highly effective in reducing the number of HIV particles in the blood stream (as measured by a blood test called viral load) and delaying disease progression. Clinical trials and observational studies have shown profound reductions in morbidity and mortality in patients infected with HIV as a result of combination antiretroviral therapy [16, 19–27]. Of relevance, advances in HIV treatment have had a positive impact on all the affected demographic and behavioral risk groups, with an expected longevity for HIV-infected patients that is now 73 years [23]. Moreover, it should be considered that, thanks to the recent expansion in the number of antiretrovirals and antiretroviral classes, virological suppression has become achievable in most patients for whom numerous prior antiretroviral regimens had failed. In addition, antiretroviral therapy results in efficacious treatment of HIV-1, regardless of the viral subtype.

However, despite advances in antiretroviral therapy, some treatments still fail. A major cause of treatment failure is the development of drug resistance both in HIV-1 B and non-B subtypes [28–34]. The extreme variability and the high evolution rate of HIV-1 favour the development of antiretroviral resistance. Indeed, HIV-1 infection is characterized by a high degree of genetic variability within infected persons. This is explained by the fact that the virus population present at a certain time point within an infected person consists of a complex mixture of heterogeneous strains, termed “quasispecies” [35]. The heterogeneity of quasispecies is due to their different antigenic and phenotypic properties. They continuously compete among themselves for survival and propagation [36]. The subsequent overgrowth or dominance of a certain viral strain over another is largely determined by its relative adaptation to a given intrahost environment, a factor particularly relevant to the emergence of drug resistant variants. Indeed, the intrapatient virus population is a highly dynamic system, characterized by a high turnover rate and a high mutation rate [37, 38]. These evolutionary dynamics are the basis for a diversified population that can quickly generate drug-resistant variants in response to the therapy [39–42]. Escape mutants that have a selective advantage under therapy become dominant in the population and lead to an increasing virus production and eventually to therapy failure. The shifted population may be hit with a new drug combination, but finding such a potent regimen after treatment failure is challenging, since many accumulated mutations confer drug resistance not only to the administered drugs but also to structurally and functionally similar compounds [41, 43].

Identifying and understanding HIV-1 drug resistance therefore helps clinicians to avoid minimally active antiretrovirals in favor of newer drugs that are fully or nearly fully active [44–46]. For this reason, resistance testing has become an important diagnostic tool in the management of HIV infections [47–52]. With the aid of HIV resistance tests, antiretroviral treatment strategies can be improved. Pharmacoeconomic studies have shown that these tests are also cost effective [53, 54]. For several years, national and international HIV treatment guidelines have been recommending the use of resistance testing both in drug-naive and drug-treated patients [48–52].

Antiretroviral drug design, resistance research, and interpretation systems have been largely based on HIV-1 subtype B because of its predominance in the wealthy countries in which antiretroviral drugs were first introduced, as well as the availability of assays for drug resistance in such locations. However, it should be noted that HIV-1 B subtype represents only about 10% [55–57] of the overall subtypes in the world. Several studies showed that non-B subtype HIV-1 infections have been rapidly increasing over time in previously subtype B homogeneous areas such as Europe and North America [58–73]. For example, in France, Switzerland, and Italy, it is estimated that non-B infections constitute roughly 15%–35% of HIV-1 infections, with an increasing prevalence and complexity of intersubtype recombinants over the last years [59, 64, 66, 73–77]. Although non-B infections are infrequent in North America, a study in New York identified non-B infections in a few US citizens who never travelled abroad, suggesting that transmission of non-B subtype occurs in the United States independently of travel history [72]. Moreover, new HIV-1 strains are constantly emerging [78, 79].

Due to the spread of HIV-1 non-B subtypes and the introduction of antiretroviral drugs in resource-limited settings (known for the largest assortment of non-B subtypes), further knowledge concerning the responsiveness to antiretroviral therapy and HIV-1 drug resistance in non-B subtypes is required. In this regard, the development of specific mutations varies in different subtypes, and this can be explained mainly by the intrinsic properties of the virus and not only by a different pressure of antiretroviral drugs, as suggested in a recent study [76]. To date, the improvement of the genotypic resistance test in terms of sensitivity, cost effectiveness and detection of drug resistance in non-B subtypes is a major topic. In order to achieve this goal, the introduction of new affordable assays is crucial. Group M subtype independent genotyping assays for detection of HIV-1 drug resistance were recently developed. These assays could represent an alternative to commercial assays for HIV-1 drug resistance genotyping in routine diagnostics and for surveillance and monitoring of drug resistance in resource limited settings [80–82].

Several discrepancies are still present in the interpretation of resistance patterns present in HIV-1 non-B subtypes by using different interpretation algorithms. These discrepancies illustrate how hard it may be to reach an unambiguous conclusion, despite the efforts made over the last decade to interpret drug resistance in HIV-1 non-B subtypes [83–87]. Further and continuous refinement of interpretation algorithms is required to improve their prediction.

The increasing prevalence of HIV-1 non-B subtypes could have implications not only for the development of resistance but also for diagnosis [88], vaccine design [89], and the clinical management of HIV infection [65, 90, 91]. Moreover, patients infected by certain HIV-1 non-B subtypes present accelerated disease progression [92–97] and higher cognitive impairment [98].

In the light of the above mentioned, the aim of this review is to provide a complete picture about HIV-1 genetic diversity and its implications.

## 2. HIV-1 Genetic Diversity

HIV-1 is characterized by extensive genetic diversity. Mutational escape results in a remarkable degree of viral diversity within HIV-1 and in its adaptation to both immune activity and antiretroviral therapy. However, not all escape mutations are advantageous to the virus since some of them can severely affect viral fitness [99, 100]. The extensive genetic diversity of HIV-1 is due to its high replication rate, the error-prone reverse transcriptase, and recombination events that may occur during virus replication [101, 102].

### 2.1. Error-Prone Reverse Transcriptase Enzyme and High Replication Rate

The molecular basis of HIV-1 variability is a highly error-prone reverse transcriptase enzyme [103]. The activity of this enzyme, essential for viral replication, is specifically required for the conversion of single-stranded genomic RNA into double-stranded viral DNA, which is later integrated into the host genomic DNA [104]. For this reason, HIV-1 reverse transcriptase inhibitors are powerful inhibitors of HIV-1 replication and represent an important class of antiretroviral agents [105]. HIV-1 reverse transcriptase is a multifunctional enzyme that possesses RNA-dependent and DNA-dependent DNA polymerase activities as well as an RNase H activity that specifically degrades the RNA strand of RNA/DNA hybrids [104]. As an intrinsic property, and in contrast to other DNA polymerases, HIV-1 reverse transcriptase lacks a proofreading function. This error-prone nature of reverse transcriptase, together with the high rate of virus production sustained by HIV-1 infection *in vivo*, strongly contributes to the continuous generation of new viral variants [13, 106, 107].

The rate of nucleotide substitutions introduced by reverse transcriptase is approximately 10^−4^ per nucleotide per cycle of replication, which is equal to one nucleotide substitution per genome during a single replication cycle [108]. Insertions, deletions, and duplications also contribute to the genetic heterogeneity of HIV-1 [109].

HIV-1 has a rapid turnover, and it is estimated that approximately 10^9^ virions per day are generated in an infected individual. The composite lifespan of plasma virus and virus-producing cells is very short with a half-life of approximately two days, and an almost complete replacement of wild-type strains by drug resistant virus occurs in plasma within 2–4 weeks [107]. During antiretroviral treatment, rapid viral turnover in combination with a high mutation rate is a primary factor behind the emergence of HIV variants with antiretroviral drug resistance.

### 2.2. Genetic Recombination

Genetic recombination is another important strategy by which HIV generates genetic diversity [109]. This process contributes strongly to high level multiple drug resistance [110–112]. Each retroviral particle contains two copies of single-stranded RNA, and template switches occur frequently during reverse transcription, thus generating mutations and recombination by intramolecular and intermolecular jumps. Recombination may link drug resistant mutations in HIV-1, leading to increased resistance to a particular drug [113], or the generation of multidrug resistant variants [110]. In addition, recombination may lead to the acquisition of mutations that compensate for a loss in viral fitness or replicative capacity due to previous acquisition of resistance mutations.

Since recombination can create a multiple drug resistant virus out of two single drug resistant strains, it is generally believed that the capacity of the virus to recombine facilitates the evolution of drug resistance [9, 110, 113–115]. This rapid evolution of drug resistance in HIV remains a major obstacle for HIV therapy.

Recombination is a strategy for viral rejuvenation, and it is likely that recombination between HIV strains may lead to the evolution of fitter forms and viral strains acquiring drug resistance to all major classes of HIV-1 inhibitors. A different scenario could be that a fitter virus can be generated by recombining parts of two parental genomes with lesser fitness, or alternatively a less fit virus can be generated by breaking up favourable combinations of mutations in the parental genomes. This interaction between recombination, mutations, and viral fitness is highly intricate, but nonetheless, recombination and its mechanisms, especially at the level of diverse subtypes, warrant further investigation. The potential for genetic differences among subtypes to yield different patterns of resistance-conferring mutations is supported by natural variation among HIV subtypes in genetic content (40% variation in the *env* gene, and 8–10% variation in the *pol/gag* genes). This issue acquires special relevance in view of the fact that the HIV *pol *gene is the major target for all major classes of anti-HIV drugs and most HIV strains show hotspots for recombination in *gag-pol* and *env* regions.

## 3. Origin of HIV-1 and Its Distribution

On the basis of genetic homology, HIV-1 has been classified into four groups: M (main), O (outlier), N (non-M, non-O), and the recently identified P [9–12]. The M group further splits into 9 subtypes (A through J) [13–15], as well as at least 58 circulating recombinant forms (CRFs, http://www.hiv.lanl.gov/content/sequence/HIV/CRFs/CRFs.html, last accessed 06 May 2013) and multiple unique recombinant forms (URFs). In general genetic variation is of 25–35% and 15–20% between and within subtypes, respectively. The genetic variation within some subtypes can determine distinct sequence clusters. For example, subtypes A and F have been subdivided into five (A1, A2, A3, A4, A5) and two subtypes (F1, F2), respectively.

CRFs are intersubtype recombinant HIV-1 genomes that have infected three or more subjects who are not epidemiologically related, so they can be assumed to make an epidemiologically relevant contribution to the HIV-1 M group epidemic. The CRFs are labelled with numbers rather than letters, and numbered in the order they were first adequately described as the first time. For example, CRF02_AG is the second CRF that has been described [116]. URF variants are widely distributed worldwide, with recombination breakpoints different from those found in CRFs.

Subtypes within the HIV-1 N and O groups are not yet clearly identified. Very few isolates of HIV-1 N group have been identified and sequenced from humans, while the diversity of sequences within the O group is nearly as great as the diversity of sequences in the M group.

Today, the HIV-1 M group, the cause of the worldwide pandemic, has a near global distribution, whereas the N and O groups are restricted to individuals of West African origin. The P group was recently identified in two individuals originating from Cameroon. The M and N HIV-1 groups have a common ancestor, one that is most closely related to the SIV strain found in chimpanzees (SIVcpz; *Pan troglodytes troglodytes*) [117, 118] that live mainly in Gabon, Cameroon, and the Republic of the Congo. This HIV-1 progenitor probably was passed from chimpanzees to human hunters through blood borne transmission [119, 120]. Recent evidence suggests that the HIV-1 O and P groups may have originated in wild gorillas, in which the closest relatives of these two groups have been identified [10, 121].

Phylogenetic analysis of HIV-1 and related viruses from nonhuman primates suggests that three independent transmission events, which happened in early 20th century, spawned the HIV-1 M, N, and O groups [119, 120]. In particular, the earliest direct evidence of HIV infection in humans was found retrospectively in a serum sample and in a lymph node biopsy specimen stored in 1959 and 1960, respectively, in Kinshasa in the Democratic Republic of the Congo [122, 123]. These samples provided direct evidence of the age of the HIV epidemic in humans. Moreover, they have been instrumental in the validation and extrapolations of molecular clock computer programs used to estimate the time to the most recent common ancestors (tMRCAs) and evolutionary rates of various HIV lineages [123, 124]. The HIV-1 M group appears to be the oldest HIV lineage in humans, with an estimated time to the tMRCA in the first two or three decades of the 1900s [123, 124]. In particular, the tMRCA shared between the M group and SIVcpz is estimated at 1853 (1799–1904) [125]. Cross-species transfer is therefore inferred to have taken place sometime between 1853 and the early 1900s, although there is some uncertainty, given the size of the confidence intervals.

HIV-1 M group subtypes can be considered as a result of the high error rate of reverse transcriptase enzyme during virus replication and selective pressure exerted by the immune system. Since their introduction into humans, HIV-1 M group subtypes have expanded rapidly in West and Central Africa and established multiple epidemics around the world. Regional expansion of HIV-1 has come in waves with the rapid emergence of specific subtypes due in part to specific modes and routes of transmission. For example, intravenous drug use in Southeast Asia in the mid-1980s and in Eastern Europe and Russia during the early 1990s led to the rapid spread of CRF01_AE and of subtype A, respectively [126, 127]. A similar expansion of subtype B HIV-1 transmission occurred among men who have sex with men in North America and Europe in the early 1980s. However, HIV-1 subtype C (the most dominant subtype in the world responsible for more than 50% of overall infections) appears to have slowly emerged throughout the world over the past 10 to 15 years as a consequence of multiple introductions [126, 128].

In recent years a substantial increase of recombinant forms has been observed as a consequence of the increased genetic complexity of the global epidemic [57, 59, 64, 66, 73–77, 129–131].

However, the prevalence of recombinant forms (estimated to be around the 20% [57]) is still underestimated. Indeed, genetic complexity is not always detected, and this is mainly due to the subtyping of only one genetic region and not of the full genome. Consequently, specimens previously considered “pure” variants may be classified as recombinants when additional viral genes are analyzed.

## 4. Development of Resistance to Antiretroviral Therapy among Different HIV-1 Subtypes

**Figure 1:**
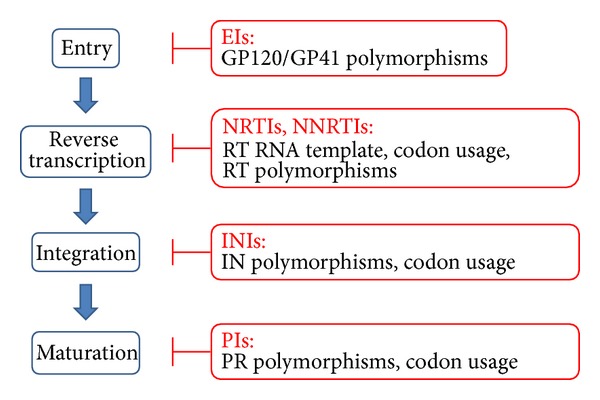
Steps of HIV-1 life cycle targeted by antiretroviral drugs and relative impact of HIV-1 subtype on resistance development. Blue boxes represent the crucial steps of HIV-1 life cycle targeted by antiretrovirals. Red boxes report the drug classes available in clinics and the HIV-1 genetic characteristics related to subtype potentially involved in resistance development. EIs: Entry inhibitors; IN: integrase; INI: integrase inhibitors; NRTIs: nucleotide reverse transcriptase inhibitor; NNRTI: non-NRTIs; RT: reverse transcriptase; PR: protease.

The drugs currently approved for clinical use by the US Food and Drug Administration (FDA) and used to treat HIV infection belong to 6 distinct classes (http://www.fda.gov/ForConsumers/byAudience/ForPatientAdvocates/HIVandAIDSActivities/ucm118915.htm, last accessed 06 May 2013): (1) seven nucleoside and one nucleotide reverse transcriptase inhibitors (NRTIs); (2) five nonnucleoside reverse transcriptase inhibitors (NNRTIs); (3) nine protease inhibitors (PIs); (4) one fusion inhibitor (FI); (5) one CC chemokine receptor 5 (CCR5) antagonist; (6) two integrase inhibitors (INIs). Each of these drug classes act at different steps in the HIV replication cycle (Figure 1) [41]. The NNRTIs and NRTIs block the reverse transcription of the viral RNA genome in cDNA, which is catalysed by the reverse transcriptase. The PIs block protease mediated maturation of released virions. The FIs and CCR5 antagonists block HIV entry into cells. The INIs block the integration of viral genome into the DNA of the host cell.

Although it seems that combination antiretroviral regimens are effective against all HIV-1 group M subtypes, there is emerging evidence of subtype differences in drug resistance relevant to antiretroviral strategies in different parts of the world. For this purpose, extensive sampling of HIV genetic diversity and *ad hoc* analyses are required to tailoring of antiretroviral therapies in different parts of the world. HIV-1 non-B subtypes present clade-specific substitutions in positions related to drug resistance [132] that could accelerate the emergence of drug-resistant viruses, change or induce alternative pathways of resistance, influence viral replicative capacity *in vitro* [133], impair the interpretation of genotypic resistance algorithms [70, 77, 134–137], and affect drug-binding affinity [138, 139].

### 4.1. Subtype Propensities to Develop Mutations

Specific mutation development varies with different subtypes because of the intrinsic properties of the virus as well as the different pressures of antiretroviral drugs. In particular, three factors are involved in this phenomenon.

- Intersubtype differences in codon usage: Subtype differences in nucleotide and mutational motifs, which are defined as the number of transitions or transversions needed to develop resistance to different classes of antiretroviral drugs, may affect the genetic barrier for resistance, as shown in the development of some mutations in different HIV-1 proteins, such as (i) mutations associated with resistance to NNRTIs at the codon 106 of the reverse transcriptase in C and B subtypes (see Section 4.2.2) [140, 141]; (ii) mutations at the protease codons 74 in C subtypes, 82 in G subtypes, and 89 in several non-B subtypes such as C and CRF02_AG (see Section 4.2.3) [87, 142–145]; (iii) mutations in the integrase gene at codons 140, 148, 151, 157, and 160 (see Section 4.2.4) [146, 147].
- Intersubtype amino acid differences involved in minor structural changes in the targets of therapy: In this situation, different mutations emerge under the same drug pressure (e.g., the protease mutation D30N; see Section 4.2.3).
- Intersubtype differences in sequence motifs favoring nucleotide substitutions involved in drug resistance (e.g., the reverse transcriptase mutation K65R; see Section 4.2.1).

### 4.2. Impact of HIV-1 Subtypes on the Different Antiretrovirals

The following subsections describe the impact of HIV-1 subtypes on the different classes of antiretrovirals so far available.  Table 1 provides an overview of the resistance associated mutations which are related to subtype diversity.

#### 4.2.1. Nucleotide (Side) Reverse Transcriptase Inhibitors (NRTIs)

The impact of subtype in terms of emergence of resistance in the NRTI antiretroviral class is mainly due to the more rapid selection of primary resistance mutations in HIV-1 subtype C infected patients than in those infected by A subtype B virus.

**Figure 2:**
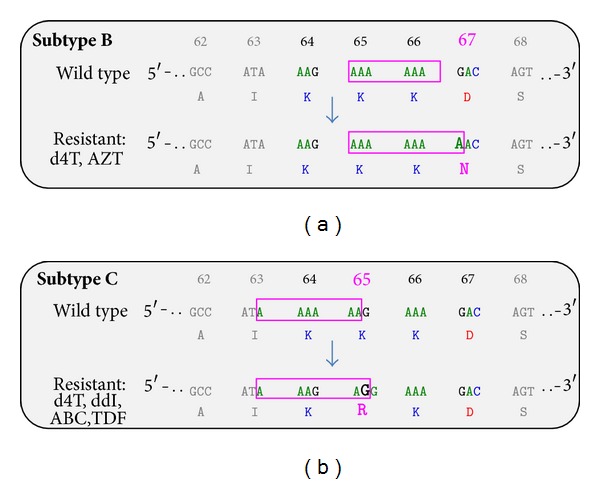
Different subtype associated RNA templates and development of NRTIs resistance in B and C subtypes. The figure represents the differences between subtype B (a) and subtype C (b), observed at nucleotide level in reverse transcriptase codons 64-65-66. AZT: zidovudine, d4T: stavudine, ddI: didanosine, ABC: abacavir, and TDF: tenofovir (modified by [15]).

Some of the differences in rates of acquisition of the mutation K65R or thymidine analogue mutations (TAMs) are doubtless due to treatment regimens and disease stage, as well as access to viral load testing in many developing countries. However, one study suggests that increased rates of K65R acquisition in subtype C may be due to the nature of the subtype C RNA template. The lysine to arginine mutation at position 65 (K65R) is a major mutation that confers broad high-level resistance to most NRTIs, except zidovudine [34]. The nucleotide sequence at codons 64-65-66 (containing a homopolymeric stretch of adenine bases) differs between subtypes B and subtype C viruses. This leads to reverse transcriptase pausing during the synthesis of double-stranded DNA from the single-stranded DNA intermediate template, a process that is template-specific but independent of the reverse transcriptase enzyme [14, 15, 148]. Subsequent misalignment of the subtype C template and primer leads to the AAG to AGG change responsible for the K65R mutation [149] (Figure 2). On the contrary, subtype B templates are prone to frequent pausing at codon 67, facilitating the generation of mutation D67N and TAMs instead of K65R (Figure 2, Table 1) [148, 150, 151].

A recent study showed a very high rate (>65%) of K65R for patients infected by subtype C who failed tenofovir-based first-line regimens in South Africa [152]. Furthermore, a rate of 7 to 15% of K65R and/or K70E mutations was observed in subtype C endemic area in patients failing regimens including stavudine, didanosine, or zidovudine [153]. Studies from Israel reported a high frequency of K65R with treatment failure in subtype C viruses from Ethiopian immigrants [154], while in Indian patients a rate of K65R around 10–12% at failure of first line combination therapy including stavudine, lamivudine, and nevirapine was observed [155]. By using ultrasensitive pyrosequencing, variants carrying K65R mutation showed a higher frequency in subtype C infected patients than those infected by subtype B (1.04% versus 0.25%). However, these results were not duplicated using the limiting dilution clonal sequencing approaches [156].

In addition to the recruitment for monitoring K65R prevalence in patients with subtype C HIV infection, also the TAM pathway (67N/70R/215Y) deserves further attention. Indeed, in patients infected by subtype C from Botswana, India, South Africa, and Malawi TAMs were also observed at treatment failure with zidovudine and didanosine [153, 155, 157, 158]. Larger numbers of patients and followups will be required to determine whether any consistent effect of the emergence of K65R in subtype C is clinically relevant.

#### 4.2.2. Nonnucleotide Reverse Transcriptase Inhibitors (NNRTIs)

The emergence of mutations associated with resistance to NNRTIs occurs after a single dose of nevirapine (sdNVP) [159–161], a procedure recommended for the prevention of mother-to-child HIV transmission (PMTCT) by initial guidelines from the World Health Organization (WHO) [162, 163]. Replacement of sdNVP may be necessary to reduce infant HIV infection risk and drug resistance development. Indeed, high frequency of drug resistance was observed in 69% of pregnant women infected with subtype C, 36% of those with subtype D, 19% of those with subtype A, and 21% of those with the CRF02_AG, despite the absence of resistance before the administration of antiretroviral therapy [163–167]. Moreover, by using ultrasensitive sequencing techniques, several studies revealed that in patients infected by subtype C a higher prevalence of nevirapine-resistance mutations (K103N and Y181C) was found, reaching 70% to 87%, as compared with 42% of patients with subtype A with resistant viruses [168, 169]. These findings underscore the role of viral subtype in facilitating the development of drug resistance, which is exacerbated by the fact that resistance to NNRTIs can also be transmitted through breast feeding [170].

The propensity for subtype C viruses to develop resistance also affects the NNRTI class. Indeed, the V106M mutation is commonly selected in subtype C viruses (in approximately 30% of patients) after exposure to nevirapine or efavirenz, whereas a V106A substitution is only rarely selected (in approximately 5% of patients) by these two NNRTIs in other subtypes (Table 1).

This peculiarity of subtype C results from the fact that V106 is encoded by GTA in subtype B viruses and GTG in subtype C viruses [141, 171]. A single G-to-A transition at the first position of codon 106 in subtype C viruses results in V106M, which confers high-level resistance to efavirenz and nevirapine. In contrast, in subtype B viruses, V106M requires two nucleotide substitutions (GTA-ATG) and therefore occurs infrequently [140, 141].

K103N and Y181C mutations remain important in both subtypes B and C, with K103N occurring in 40% of subtype B and 29% of subtype C viruses and Y181C occurring in 23% of subtype B and 12% of subtype C viruses [172]. In a recent Italian study, the prevalence of mutation K103N in NNRTI-treated patients was lower in subtype C infected patients compared with patients infected by B, F, or CRF02_AG subtypes (Table 1). Conversely, the mutations Y181C and Y188L were found with higher frequency in patients infected with subtype C compared to patients infected with subtype B [76].

Another substitution that is seen more frequently among patients with a subtype C virus is G190A, which is also a result of naturally occurring G190A/S polymorphisms [140]. Reverse transcriptase polymorphisms at residue 98 are common in subtype G, but they are also associated with NNRTI resistance in subtype B (Table 1) [15, 173].

The second-generation NNRTI etravirine has demonstrated good efficacy across subtypes [174]. A recent study highlighted the fact that HIV-1 genetic variant does not significantly influence the genotypic prediction of etravirine susceptibility in infected individuals failing efavirenz containing regimens [175]. However, among the etravirine resistance associated mutations, some polymorphic substitutions resulted in drug-naive individuals infected with non-B subtypes, especially CRF02_AG [176]. Noteworthy, in a recent study it was found that the mutation E138K was the first mutation to emerge in either subtype B, C, or CRF02_AG clinical isolates under etravirine pressure (Table 1) [177]. Another study confirmed this finding for subtype C, while a preferential selection of Y181C for the subtype B virus was observed (Table 1) [178].

A novel mutation in the C-terminal domain of reverse transcriptase (N348I) has recently been reported to reduce susceptibility to etravirine in subtypes A, B, and C (Table 1) [179]. In particular, in a South-African clinical trial, the N348I mutation was observed in 24% of subtype C infected patients failing a first-line NNRTI regimen (most commonly with nevirapine) [180]. This mutation is not included in standard mutation lists or algorithms, but more data are urgently required to determine its clinical relevance.

To date, the efficacy and the resistance patterns of the novel second-generation NNRTI rilpivirine seem to be similar across the different subtypes [181, 182].

A study focused on HIV-1 subtype CRF01_AE showed that the presence of mutations associated with resistance to rilpivirine are uncommon in patients infected by this subtype and failing a first-line NNRTI-containing regimen [183]. However, cross-resistance between rilpivirine and etravirine was high. Moreover, approximately 60% of patients had a high level of etravirine resistance, thus suggesting that the role of etravirine in HIV-1 subtype CRF01_AE infected patients in second-line therapy is limited in late NNRTI failure settings [183].

NNRTI resistance associated mutations such as Y181I, Y188L, G190A, K101A, V106I, and V179I are natural polymorphisms in HIV-2, thus HIV-2 proves to be naturally resistant to all NNRTIs [184].

#### 4.2.3. Protease Inhibitors (PIs)

Nonpolymorphic substitutions in the protease gene have a greater impact on baseline susceptibility to PIs than polymorphic mutations [185]. However, intersubtype amino acid differences can create subtle structural differences in the targets of therapy. For example, subtype B infected patients receiving nelfinavir are more likely to develop D30N than those with viruses belonging to subtypes C, F, G and CRF01_AE, which are more likely to develop L90M or N88S (Table 1) [186, 187]. More specifically, by comparing B and G subtypes, even in cases where the first mutation is L90M in both these subtypes, subsequent mutations differ significantly. Indeed, in subtype B, L63P is the second mutation and occurs in almost all cases, suggesting that the progression of resistance is dependent on the emergence of this mutation, followed by the selection of V77I and other mutations. Differently, in subtype G, in almost 100% of cases, L89I follows the emergence of L90M, suggesting a role in subtype G similar to that of L63P in subtype B. In this regard, findings indicate that the association of L89I with L90M may further increase the PI resistance of subtype G viruses when compared with L90M alone [188]. The third emerging mutation can be either A71V or I54V.

Although subtype C isolates from Ethiopian immigrants to Israel favoured the L90M pathway, patients with subtype C viruses in Botswana did have D30N, suggesting that subtype C viruses from Ethiopia and Southern Africa might be different [186, 189]. The basis for the higher preponderance of D30N in subtype B than in other subtypes may be related to the flexibility of the protease flap region and destabilization of the PI complex in subtype B, whereas an accessory N83T mutation may be needed to rescue fitness and confer resistance in subtype C [142, 190].

Recent evidence has suggested that the polymorphism at codon 36 in the protease gene (M36 in subtype B and I36 in most other non-B subtypes) affects both the patterns of resistance that emerge under drug pressure and viral replication capacity (Table 1) [191]. Similarly, the M89 polymorphism in subtypes A, C, and CRF01_AE (L89 in subtype B) preferentially leads to the emergence under drug pressure of the M89T mutation, which confers high-level resistance to nelfinavir, atazanavir, and lopinavir [192]. There is also *in vitro* evidence that CRF2_AG viruses with the 17E/64M polymorphisms demonstrate hypersusceptibility to certain PIs (nelfinavir, atazanavir, and indinavir) [193].

Differences in polymorphisms in the protease gene have been reported among several non-B subtypes; these include the following protease residues: 10, 20, and 63 in subtype A; 20, 53, 63, 74, and 82 in subtype C; 13 and 20 in subtype D; 10, 14, 20, and 77 in subtype F; 20, 67, 73, 82, and 88 in subtype G; 20, 63, 82, and 89 in the CRF01_AE; 20 and 89 in the CRF02_AG (Table 1) [194]. In particular, in patients treated with PIs, L89V was predominantly found in CRF02_AG, while the tipranavir resistance mutation T74P was predominantly found in the C subtype in comparison to B subtype [76]. The mutation V82M was mainly found in subtype G while the mutations V82A/F/S were mainly present in other subtypes; the mutation N88S was found in subtypes C and CRF02_AG, while N88D was present in subtype B [194]. In southern Brazil, scientists reported a lower relative frequency of primary resistance to PIs in subtype C rather than in subtype B [195].

Despite clear evidence for preferential emergence, subtype diversity may not limit the initial benefits of antiretroviral therapy [196].

Diminished susceptibilities among wild-type isolates have been found for CRF02_AG recombinant viruses in three different studies of nelfinavir and atazanavir [138, 173, 194]. A study suggested that distortions in the K26 pocket of the A/G protease may be responsible for a lower binding energy of nelfinavir and lower susceptibility of A/G viruses [138].

The protease and *gag* genes coevolve as a functional unit when HIV is subjected to drug pressure from PIs. Mutations in *gag *can act as compensatory substitutions that can increase both rates and levels of resistance to PIs, as well as viral replication capacity [197]. No genotypic system for the determination of drug resistance to PIs currently monitors *gag*, despite the fact that relevant mutations in *gag* can increase resistance by a factor of 2 to 2.5, depending on the subtype. It is likely that different subtypes could develop compensatory *gag *mutations at different rates; this might ultimately justify the genotyping of *gag *in resistance testing.

#### 4.2.4. Integrase Inhibitors (INIs)

The primary mutations in the integrase gene associated with INI resistance are E92Q, Y143R/C, Q148K/R/H, and N155H. The residues associated with primary resistance seem to be highly conserved across subtypes, but polymorphisms at the sites of secondary resistance mutations are more common in non-B subtypes [147, 198–200].

Signature subtype differences in integrase at codons 140, 148, 151, 157, and 160 among HIV subtypes may affect the genetic barrier for resistance [146]. These variations predict higher genetic barriers to the development of G140S and G140C mutations in subtypes C, CRF02_AG, and A/CRF01_AE, as well as higher genetic barriers to V151I in subtypes CRF02_AG and CRF01_AE [146, 147].

Regarding the impact of subtype on primary resistance development, it was recently observed that the subtype B integrase enzyme with the N155H mutation (±E92Q) exhibited increased resistance to raltegravir compared to the subtype C enzyme (Table 1) [201]. In addition a new potential resistance pathway for raltegravir might include the mutation G118R, a substitution associated with lower susceptibility to raltegravir in CRF_02AG subtypes [202].

Notably, polymorphisms such as T97A, V151I, and G163R, already known as raltegravir secondary resistance mutations, showed a considerable prevalence (≥9%) in several recent studies also analyzing non-B subtype infected patients [203, 204]. The additional INI-resistance associated mutations K156N and S230N mutations were more frequent in B subtype while V72I, L74I, T125A, V201I, and T206S were more frequent in certain non-B subtypes [200, 205]. No differences in phenotypic raltegravir resistance were found in several non-B isolates tested [196].

Dolutegravir is a second-generation INI which recently entered the battery of antiretrovirals against HIV-1 infection. It showed a higher genetic barrier to resistance and retains activity against viruses with resistance to raltegravir or elvitegravir. Interestingly, among the mutations associated with *in vitro* resistance to dolutegravir, the mutations L101I and T124A were polymorphic and significantly more prevalent in patients with non-B subtypes [200]. In addition, mutation R263K seems to be preferentially selected in subtype B under dolutegravir pressure (Table 1) [206].

#### 4.2.5. Entry Inhibitors (EIs)

Among the EIs today available in clinics, both enfuvirtide (targeting gp41 HIV-1 protein) and maraviroc (targeting cellular CCR5 receptors) have shown antiviral activity against HIV-1 B and non-B subtypes [207, 208]. However, the high level of diversity (20 to 40%) in the *env* region predicts that this class of drugs will probably have higher potential for natural and emergent drug resistance. Clinical data have shown that the FI enfuvirtide is active against non-B subtypes owing to a highly conserved binding domain, although HIV-2 and HIV-1 class O viruses have natural resistance against this drug [209, 210]. So far, some studies suggest that there are significant differences in baseline susceptibility to HIV EIs among the predominant HIV-1 subtypes [211, 212]. For instance, it was found that in drug-naive patients many more mutations associated with resistance to EIs occur in subtype C compared with subtype B strains (Table 1) [211].

## 5. Clinical and Biological Relevance of HIV-1 Genetic Diversity

Due to the worldwide spread of non-B viruses and the introduction of antiretroviral drugs in developing countries (known for the largest assortment of non-B subtypes), further knowledge concerning responsiveness HIV-1 drug resistance in non-B subtypes is required. Moreover, it should be taken into account that the increasing prevalence of HIV-1 non-B subtypes could have several implications not only for the development of resistance but also for other topics such as the response to antiretroviral therapy, disease progression, and transmission.

### 5.1. Impact of HIV-1 Subtypes on the Drug Resistance

There is a solid body of evidence indicating that the type and degree of HIV-1 resistance to NRTIs, NNRTIs, and PIs vary between different subtypes [136, 186, 213, 214]. The development of nelfinavir resistance in subtypes B and G represents a classic example of this phenomenon. A different level of resistance has been observed among different subtypes. Indeed, the recombinant form CRF02_AG is more susceptible to nelfinavir and ritonavir than subtypes C and F; subtype G is more sensitive to tipranavir and lopinavir than other subtypes [135], and the subtype C has accelerated risk in developing resistance to tenofovir [189, 215, 216].

An explanation for the extreme variability of HIV-1 subtypes in the response to antiretrovirals can be given by the presence of some polymorphisms that can influence both the emergence of drug-resistance mutations and the response to drugs. For example, polymorphisms at residues 20 and 36 of HIV-1 protease decrease the genetic barrier to tipranavir resistance in subtypes A, C, F, and G [133], while nucleotide heterogeneity at 64 and 65 positions in the reverse transcriptase accelerates development of K65R in subtype C [150, 189]. In some cases, drug exposure may lead to amplification of such polymorphisms as A98G/S in reverse transcriptase and M36I, K20I, and L89M in protease, leading to a potential for resistance [139].

It is commonly accepted that, as a result of a high degree of polymorphism in the protease gene, drug-naive patients infected with non-B subtypes present a certain degree of resistance to the PIs. However, the data which support this assumption are largely controversial, as highlighted by two studies that showed conflicting results. Indeed, in a study [143] analysing the HIV-1 phenotype from 42 patients (19, subtype G; seven, subtype C; six, subtype F; 10, CRF02_AG) by the Antivirogram Assay (Virco BVBA), no differences in baseline susceptibility to any PI were found, with the exception of an unexpected hypersusceptibility to nelfinavir with CRF02_AG. On the contrary, a second study, investigating drug susceptibility of 39 isolates from treatment-naive Ghanaian patients [138], mostly infected with CRF02_AG, showed reduced susceptibility to several PIs, especially nelfinavir. This reduced *in vitro* susceptibility is not in agreement with the available clinical data, which show no difference in time to undetectable viral load among different subtypes [217–219].

Continuous research on the role of polymorphisms in the development of drug resistance is therefore necessary. Studies are needed to assess genotypes both before and after therapy in the context of possible associations between polymorphisms and drug resistance. This area of research could include polymorphism variability in variants of the same subtype in different geographic regions. This information might improve the efficacy of certain drug combinations over others in the context of second- or third-line therapeutic strategies. Polymorphisms should improve current algorithms for interpretation of genotyping results in a subtype-independent way.

As access to antiretroviral therapy in resource limited settings increases, where non-B subtypes are predominant, it remains imperative to establish appropriate treatment strategies for long-term clinical benefit that limits the emergence of drug resistance. The use of nontoxic, effective antiretroviral drugs should yield excellent clinical responsiveness, regardless of the viral subtype. Subtype differences, suboptimal therapies, and deficiencies in health care delivery systems can create conditions for accelerated development of resistance. Urgent recruitment for low-cost viral-load monitoring is needed to prevent and detect drug resistance, as well as to avoid unnecessary treatment switches [220, 221].

Unfortunately, pooling of resistance data often masks the role of regional differences in viral subtypes and antiretroviral therapies in the development of drug resistance. In resource-poor settings, such studies have used different NRTI backbones (e.g., combinations of zidovudine and didanosine, zidovudine and lamivudine, or stavudine and lamivudine). In parts of Africa, treatment failure has been reported in as many as 40% of patients after 2 years [222]. Of note, resistance rates in India to two drug classes were observed in more than 80% of patients who failed their first-line regimen using combinations of NRTIs and NNRTIs [223].

Larger longitudinal studies are necessary to determine the response to first-line combinations of antiretroviral drugs. The availability of genotypic resistance testing needs to be expanded to include all countries in which antiretroviral drugs are used.

Cross-resistance among drugs is important, especially in settings where treatment options may be limited. Relatively few *in vitro* comparative data are available for PIs in non-B subtypes, even if such data may be crucial for an understanding of cross-resistance to this class of antiretrovirals [191, 224]. Such data are important, since PIs are often the only available option for drug sequencing in resource-limited settings after the failure of first- or second-line treatment. Noteworthy, PIs are drugs with a high genetic barrier. This means that resistance to PIs usually requires the presence of large numbers of resistance mutations. For this reason, there is an advantage of using PIs to avoid early resistance development. Therefore, differences among subtypes with regard to drug resistance are likely to be more important for NRTIs, NNRTIs, and INIs than for PIs.

### 5.2. Impact of HIV-1 Subtypes on Response to Antiretroviral Therapy

Our knowledge of the impact of HIV-1 subtypes on responses to antiretroviral therapy is very limited. Most studies have been small or poorly designed, and only a few have had an evaluation period longer than 48 weeks. An important limitation of these studies was to compare subtype B with non-B subtypes grouped together, a necessary oversimplification when dealing with small numbers of patients infected with non-B subtypes [217–219, 225–227]. Moreover, no prospective, randomised trials specifically designed to address this question exist. A study by the Paediatric European Network for Treatment of AIDS (PENTA 5 trial) [219] investigated the response to therapy of 113 children infected with 7 different subtypes (A, 15%; B, 41%; C, 16%; D, 9%; F, 5%; G, 2%; H, 1%) and the two CRFs CRF01_AE (5%) and CRF02_AG (7%). No significant differences were observed at 48 weeks of treatment. However, these results should be used with caution because the study did not have the statistical power to rule out minor differences between subtypes. Moreover, only four drugs were used (zidovudine, lamivudine, abacavir, and nelfinavir). Although polymorphisms at resistance-associated positions were highly prevalent in non-B subtypes, no negative impact of the mutations in virological responses was found; therefore, the authors concluded that polymorphisms at resistance-associated positions in subtype B could not necessarily be interpreted as conferring resistance in non-B subtypes.

Frater et al. retrospectively analysed the virological response in 362 patients: 265 Europeans infected with subtype B and 97 Africans infected with non-B subtypes [218]. Subtype was presumed from ethnic and epidemiological data, with confirmation further extrapolated from genotyping the samples from 60% of the Africans and 30% of the European patients. There was a significant imbalance between the two groups in several important parameters, including gender, transmission-risk groups, CD4 cell counts, and antiretroviral regimens used. Results showed no difference in time to undetectable viral load or recovery of CD4 count; however, a significant difference was observed in viral load over time, with a continuous increase in the African group after 9 months, suggesting a higher rate of therapy failure. Based on indirect evidence (i.e., the number of resistance mutations at time of failure), the authors concluded that the difference in virological response was mainly due to poorer adherence in the African group rather than the infecting viral subtype. As the study was not controlled for adherence, its conclusions remain controversial.

A study which analysed retrospectively B and non-B subtypes (mainly focused on pure subtype A or recombinants with a subtype A protease) showed no differences in the time to undetectable viral load or in the viral load measured over time [217]. However, the gain in CD4 count was significantly lower in the non-B group, which showed an increase of 161 cells/mm^3^, compared with 236 cells/mm^3^ in the subtype B group. These results were consistent with those of Camacho, who compared retrospectively the pathways to nelfinavir resistance in 101 patients (46 subtype B, 55 subtype G) failing a first-line regimen including this PI [228]. At the time of failure, the only parameter significantly different between the two groups was the CD4 count (423 cells/mm^3^ in the subtype B group and 360 cells/mm^3^ in the subtype G group; *P* < 0.001). No explanation was given for this observation.

Geretti et al. assessed virologic and immunologic responses to starting HAART in a large cohort of 2116 patients, with the specific objective of comparing outcomes in patients with subtype B infection and those with subtype C, subtype A, CRF02_AG, and subtype D infection, the predominant non-B subtypes circulating in the United Kingdom [229]. Overall, 1906 (90%) patients achieved viral load undetectability within 12 months after they started HAART, of whom 335 (18%) subsequently experienced virologic rebound. In adjusted analyses, viral load suppression occurred more rapidly in patients infected with subtype C (hazard ratio (95% confidence interval, CI) 1.16 [1.01–1.33], *P* = 0.04) and subtype A (1.35 [1.04–1.74], *P* = 0.02) relative to subtype B infection. The virologic rebound occurred marginally faster in patients with subtype C infection (1.40 [1.00–1.95], *P* = 0.05), but the hazard of virologic rebound was similar with other subtypes. Although patients infected by subtype B viruses showed higher baseline CD4 cell counts and maintained the advantage throughout therapy, CD4 cell count recovery occurred at similar rates with all subtypes. These findings suggest that HAART achieves good outcomes regardless of the infecting subtype.

The effect of pretreatment HIV-1 drug resistance on the response to first-line combination antiretroviral therapy in Sub-Saharan Africa has recently been evaluated in a large prospective cohort [230]. Pretreatment drug resistance results were available for 2579 (94%) of 2733 participants. Among them, 123 (5%) had pretreatment drug resistance to at least one prescribed drug, and 52 (2%) had pretreatment drug resistance and received fully active antiretroviral therapy. More than 50% of participants for whom it was possible to determine the subtype were infected by HIV-1 subtype C (1405 patients, 54%), while 638 (25%) harboured subtype A, 296 (11%) D, 117 (5%) A/G recombinant, 68 (3%) G, 48 (2%) other recombinants, seven (<1%) other subtypes, and five (<1%) B. Compared with participants without pretreatment drug resistance, the odds ratio (OR) for virological failure (OR (95% CI): 2.13 [1.44–3.14], *P* < 0.0001) and acquired drug resistance (2.30 [1.55–3.40]; *P* < 0.0001) was increased in participants with pretreatment drug resistance to at least one prescribed drug but not in those with pretreatment drug resistance and fully active antiretroviral therapy. CD4 cell count increased less in participants with pretreatment drug resistance than in those without (35 cells per *μ*L difference after 12 months; 95% CI 13–58; *P* = 0.002). HIV-1 subtype was not associated either with acquired drug resistance or virological failure.

Further *ad hoc* studies are needed to better evaluate the therapeutic implications of specific non-B subtype differences in terms of long-term efficacy of different antiviral regimens.

### 5.3. Impact of HIV-1 Subtypes on Disease Progression and Viral Transmission

Several studies on disease progression showed that, among non-B subtypes, subtypes C and D were found to be more aggressive, followed by G, AE, AG, and A, the least aggressive of all HIV-1 subtypes [92–97].

In this regard, it should be noted that because the different HIV-1 subtypes are not uniformly dispersed, comparisons of virulence and transmissibility are hampered by potential confounders, such as ethnic, socioeconomic, and other epidemiological factors. The report by Kiwanuka et al. [96] concerning this topic provided a good opportunity to compare rates of disease progression associated with these different subtypes within a similar population, thanks to the cocirculation of HIV-1 subtypes A and D, and several intersubtype recombinants in the Rakai district of Uganda. In particular, Kiwanuka et al. compared rates of progression among HIV-1 seroconverters who were followed as part of the Rakai Health Sciences Program [96]. Subjects identified during 1997–2002 were followed through 2004, when antiretroviral therapy became available in Rakai district. The primary end point was the time to achievement of a CD4 cell count of *⩽*250 cells/mm^3^ or death due to AIDS. Progression to a CD4 cell count of *⩽*250 cells/mm^3^ was significantly less common among subjects infected with HIV-1 subtype A (20%), compared with subjects infected with subtype D (40%), recombinant forms (40%), or multiple subtypes (53%) (*P* = 0.03). Death from AIDS was also less common among subjects infected with HIV-1 subtype A. These differences were reflected in the longer time to AIDS onset for subjects infected with HIV-1 subtype A (8.05 years) compared with those infected with non-A subtypes (D = 6.49 years; recombinant forms = 5.57 years; multiple subtypes = 5.80 years). By multivariable models (adjusting for viral load, age, and sex), subjects infected with non-A subtypes more likely progressed to AIDS compared with those infected with HIV-1 subtype A. Similarly, subjects infected with non-A subtypes had an approximately 6–8-fold greater risk of death from AIDS. Viral factors that could explain some of the HIV-1 pathogenicity are higher *ex vivo* replicative capacity, higher genetic diversity, and CXCR4 coreceptor usage [231–233]. In this regard, a less common emergence of CXCR4-using (X4) variants was found in HIV-1 subtype A infection compared with HIV-1 subtype D infection, thus explaining the apparent lower virulence of HIV-1 A [234, 235].

A European study showed how subtype D has a fourfold higher rate of CD4 count decline, in the absence of antiretroviral therapy, compared with other subtypes in the study (A, B, C, and CRF02_AG, which had similar rates of CD4 loss), even when adjusted for baseline CD4 count [236].

Subtype D was also associated with higher rates of dementia in individuals with advanced immunosuppression if compared with subtype A [98]. A study by Kiwanuka et al. showed how in Uganda subtype A viruses have a significantly higher rate of heterosexual transmission than subtype D viruses [237]. The faster disease progression and the lower rate of heterosexual transmission of subtype D compared with subtype A can explain the changes in the proportions of subtypes A and D observed over time in Uganda and Kenya [238, 239]. In particular, a significant decrease in the prevalence of subtype D with a concurrent increase of subtype A was observed.

### 5.4. Immune Response according to Different HIV-1 Subtypes

Despite the importance of viral characteristics in determining the rate of HIV-1 disease progression, recent findings from genomic studies show that host genetic factors also play a crucial role. The genetic determinants that influence susceptibility to HIV-1 and limit AIDS vary in different populations and among individuals. Meta-analyses of large cohort studies have identified several genetic variants that regulate HIV cell entry (particularly chemokine coreceptors and their ligands with copy number variations), acquired and innate immunity (major histocompatibility complex (MHC), Killer immunoglobulin-like receptors (KIRs), and cytokines), and others (TRIM5-*α* and APOBEC3G) that influence the outcome of HIV infection [240–242]. Of the various genes that contribute toward host genetic propensity, MHC turns out to be the major contributor because it is responsible both for restriction of cytotoxic T lymphocyte (CTL) epitopes and for the emergence of CTL escape mutants. Leukocyte antigen (HLA) alleles have been shown to be associated with the rate of disease progression in Africans and Caucasians [243–245].

The interplay of viral, immune, and host genetic factors in the control of HIV-1 replication has been recently evaluated in HIV controllers [246, 247]. Similar efforts should be undertaken in diverse human populations infected with a variety of HIV-1 subtypes in order to understand fully the complex interplay of virus and host in AIDS pathogenesis.

## 6. Summary and Conclusions

HIV diversity plays a central role in the HIV pandemic; for this reason, it is today imperative that global molecular epidemiology surveillance is continued and improved using rigorous sampling strategies. The improved knowledge of the significance of non-B subtypes for resistance evolution and interpretation, in response to antiretroviral therapy, disease progression and vaccine design are becoming mandatory today not only because antiretroviral therapy is being introduced in countries where non-B subtypes are driving the epidemic but also because the number of infections by these variants is increasing sharply in previously subtype B homogeneous areas such as Europe and North America [57–69, 71–73, 248]. It is reassuring that current antiretroviral strategies appear to be effective against a broad spectrum of HIV subtypes. However, further *ad hoc* studies in larger and homogenous populations infected by different specific non-B subtypes are required to better evaluate their therapeutic implications in terms of long-term efficacy of different antiviral regimens. Further elucidation of viral polymorphisms and properties associated with transmission, viral load set point, and disease progression may lead to new approaches to disease prevention and treatment.

Efforts should be undertaken in diverse human populations infected with a variety of HIV-1 subtypes in order to fully understand the complex interplay of HIV and host in AIDS pathogenesis.

## Figures and Tables

**Table 1:** Main drug resistance mutations observed in different HIV-1 subtypes.

Drug class	Subtype	Polymorphisms or mutations or positions associated with drug resistance	Drug(s) related	Comments	References
Reverse transcriptase					

	C	K65R	d4T, ddI, ABC, TDF	Preferential selection	[14, 15, 148–152, 215, 216]
NRTI	C	K70E	d4T, ddI, AZT	High prevalence in subtype C endemic area	[153]
	B	D67N	d4T, AZT	Preferential selection	[148, 149, 151]

	G	A98S	NNRTIs	Common polymorphism	[15, 173]
	B, C, F, CRF02_AG	K103N	EFV, DLV, NPV	Lower frequency in subtype C compared to B, F, and AG subtype	[76, 168, 169, 172]
	B, C	V106M	EFV, NVP	Lower genetic barrier in subtype C in comparison with subtype B	[140, 141, 171]
NNRTI	C	E138K	ETR	Preferential selection under drug pressure in subtype C	[177, 178]
	C	G190A	NNRTIs	High frequency in subtype C	[140]
	A, B	Y181C	ETR	Preferential selection under drug pressure on A and B subtypes	[178]
	C	Y181C, Y188L	EFV, DLV, NPV	Higher frequency in subtype C	[76, 168, 169, 172]
	C	N348I	ETR	Higher frequency in subtype C at etravirine failure	[179, 180]

Protease					

	CRF02_AG	G17E, I64M	NFV, ATV, IDV	Hypersusceptibility in CRF02_AG	[193]
	B, C, F, G, CRF01_AE	D30N	NFV	Lower prevalence in C, F, G, and CRF01_AE subtypes under NFV pressure compared to subtype B	[186, 187]
	Non-B	M36I	PIs	Natural polymorphism	[133, 139, 191]
PI	A, C, D, F, G, CRF02_AG	10, 13, 14, 20, 53, 63, 67, 73, 74, 77, 82, 88, 89	PIs	Natural polymorphisms	[87, 132, 133, 142–145, 194]
	A, C, F, CRF01_AE	L89M	ATV, LPV, NFV	Natural polymorphism that may lead to the L89T mutational pathway	[139, 192, 224]
	CRF02_AG	L89V	FPV, DRV, LPV	Higher prevalence in CRFF02_AG compared to subtype B	[76, 143]
	B, C, F, G, CRF01_AE, CRF02_AG	N88S, L90M	ATV, NFV	Higher prevalence in C, F, G, CRF01_AE, and CRF02_AG subtypes compared to subtype B	[186, 187, 194]
	C	T74P	TPV	Higher prevalence in subtype C in comparison to subtype B	[76]
	Non-B	V82A/M/F/S	PIs	High prevalence in some non-B subtypes at failure	[194]

Integrase					

	B, C	N155H, E92Q	RAL, EVG	>10-fold resistance in subtype B in comparison to subtype C	[201]
	Non-B	T97A, V151I, G163R	RAL	High frequency in non-B subtypes endemic area	[203, 204]
INI	Non-B	L101I, T124A	DTG	Higher frequency in non-B subtypes in comparison to subtype B	[198]
	C, CRF02_AG	G118R	RAL	Emerging at RAL failure in subtype CRF02_AG	[202]
	B	R263K	DTG	Preferential selection under drug pressure	[206]

*Env* gene					

FI	C	N42S, L54M, A67T	T20	Higher frequency in subtype C in comparison to B	[211]
CCR5 inhibitors	C	R315Q, A316T	MVC, VCV	Higher frequency in subtype C in comparison to B	[211]

ABC: abacavir; ATV: atazanavir; AZT: zidovudine; d4T: stavudine; ddI: didanosine; DRV: darunavir; DTG: dolutegravir; ETR: etravirine; EFV: efavirenz; EVG: elvitegravir; FI: fusion inhibitor; FPV: fosamprenavir; INI: integrase inhibitor; LPV: lopinavir; MVC: maraviroc; NFV: nelfinavir; NRTI: nucleotide reverse transcriptase inhibitor; NNRTI: non-NRTI; NVP: nevirapine; PI: protease inhibitor; RAL: raltegravir; TDF: tenofovir; T20: enfuvirtide; VCV: vicriviroc.
